# Learner autonomy, learner engagement and learner satisfaction in text-based and multimodal computer mediated writing environments

**DOI:** 10.1007/s10639-023-11615-w

**Published:** 2023-04-04

**Authors:** Zohre Mohammadi Zenouzagh, Wilfried Admiraal, Nadira Saab

**Affiliations:** 1 Department of English Teaching and Translation, Karaj Branch, Islamic Azad University, Karaj, Iran; 2 ICLON, Leiden University Graduate School of Teaching, Leiden University, Kolffpad 1, 2333 BN, Leiden, Netherlands; 3 Centre for the Study of Professions, Oslo Metropolitan University, Pilestredet 40, N-0170 Oslo, Norway

**Keywords:** Learner autonomy, Learner engagement, Learner e-satisfaction, Text-based computer mediated modality, multimodal computer mediated modality

## Abstract

Technology creates variant learning experiences which are context specific. This study examined the comparative potential of multimodal and text-based Computer Mediated Communication (CMC) in fostering learner autonomy, learner engagement and learner e-satisfaction as well as learner writing quality. To this end, 40 Iranian male and female EFL (English as foreign language) students were selected on the basis of their writing proficiency and were randomly assigned into text-based and multimodal CMC research groups. Learner autonomy was investigated using Van Nguyen and Habók ‘s learner autonomy questionnaire, which had 40 items rated on 5 point likert scale, both before and after the treatment. Student engagement was tracked by analyzing transcription of stored conversations of Moodle and Discussion logs of an online writing forum, using a coding scheme to identify cognitive, emotional, and behavioral student engagement. The potential of text-based CMC and Multimodal CMC in fostering writing quality was examined by comparing students’ writing before and after treatment. Finally, students were asked to write reflective essays on their evaluation of efficacy of the learning environments. Content analysis was conducted on the open and axial coding of indicators of student satisfaction. The results of between group comparison indicated that students were more autonomous in text-based modality than in multimodal CMC. Chi-square analysis indicated that text-based CMC group outperformed multimodal CMC group in terms of behavioral and cognitive engagement. Yet, multimodal CMC group reported higher emotional and social engagement. One-way ANCOVA results also indicated that the students in text-based CMC group outperformed Multimodal CMC group in terms of writing quality. Learner e-satisfaction was examined by network mapping of open codes of student reflective essays. The study identified four categories that reflected students’ e-satisfaction: *learner dimension* (including learners’ attitude, learner internet self-efficacy), *teacher dimension* (including teacher presence, teacher digital competences), *curriculum dimension* (including curriculum flexibility, course quality, flexibility in interaction support system) and *internet dimension* (including internet quality and support system). However, internet dimension received negative judgments from both groups. The implications of the study and suggestions for further research are discussed.

## Introduction

With the advent of new technologies, learners are faced with unprecedented opportunities to conduct independent learning (Zhong, 2018) often viewed as effective in promoting autonomy in the context of the growing presence of ICT in educational systems (de Brito Lima et al., 2021). This has prompted renewed interest in identifying the features of online learning environments which students value in academic development and attainment (Barile et al., 2022). Cognitive load theory of multimedia learning proposes that human brain has a limited capacity for processing information through auditory and visual channels. This makes different learning resources to differ in the cognitive load they create and hence differ in their potential in optimizing higher quality learning (Paas et al., 2016). The pedagogical value of computer mediated learning lies in its features in expanding students views of literacy with increased semiotic resources beyond language and optimizing communication environment in the digital era (Shin et al., 2020). While previous research on the pedagogical value of features of computer mediated communication (CMC) mainly focused on the impact of CMC modalities on academic performance and gain scores confirming positive association between them (Dascalu et al., 2021; Mogus et al., 2012; Mohamadi, 2018a, 2018b), scant attention has been given to how online learning modalities moderate learner psychological attributes such as learner autonomy and student engagement. (Salikhova et al., 2020). This research, therefore, is intended to investigate the potential of text-based and multimodal CMCs in not only fostering achievement gain scores but also in promoting students’ autonomy and engagement and in turn student e-satisfaction.

As technology advances in all aspects of education, it provides plenty of opportunities for independent and self-regulated learning (Zhong, 2018). Autonomous learning entails self-generated thoughts and actions that are cyclically planned as a result of individuals’ evaluation, reflection and monitoring to enhance ones engagement in strategies to achieve personal goals (Schneider & Preckel, 2017). Learner autonomy is very context specific and can be shaped and influenced by different learning environments (Broadbent et al., 2021)Technology mediated learning resources have different potentials in leading autonomous learning (Broadbent & Poon, 2015; Broadbent et al., 2021). However, research has mainly focused on comparing online learning environments with actual class environment (Dang, 2012; Ghazali, 2020) with acknowledging supportive role of online learning platforms in promoting learner autonomy and thus leaving the analysis of potentials of different online learning modalities in fostering autonomous learning open for research.

The growing popularity of multimodal resources in technology mediated learning and teaching has highlighted learner competences and awareness of affordances available to learners in engaging them in tasks and hence exercising enhanced level of autonomy (Hauck et al., 2021).

The key to learner autonomy is learner engagement which is learners’ ability in promoting their psychological commitment to stay engaged in the learning process, to acquire knowledge and build his or her critical thinking (Dixson, 2015). Student engagement has long received educational researchers’ attention due to inconsistencies in its related research findings. Inconsistencies in research findings become more when it comes to student engagement in online learning platforms. Researchers lay emphasis on the analysis of platform access logs such as clicks, logins, and active sessions which works for classic online classes. Literature on student engagement in activity based hybrid multimodal technology enhanced learning environments is very limited (Rajabalee & Santally, 2021). Besides, research demonstrates that quality of student engagement in online courses remains mixed (Kahn et al., 2017). For example, Swartzwelder et al. (2019) indicated a positive effect of text based CMC on higher order thinking, and reflection but low learner engagement whereas multimodal CMC resulted more in learner engagement but low rate of retention. Learner satisfaction and learner engagement are interwoven (Rajabalee & Santally, 2021). Learner engagement varies as learners’ level of satisfaction varies (Martin & Bolliger, 2018). The quality and acceptance of e-learning depend largely on learner satisfaction and experiences. Students in online classes, encompass a range of difficulties such as digital literacy, technical issues, support system which if not responded properly may result in decreased learner engagement (Gillett-Swan, 2017). Students’ positive or negative perception effects how students apply knowledge and plan learning and achieve outcome and direct autonomous learning (Abdous, 2019; Mihanović et al., 2016). Therefore, the interplays between satisfactions, learner engagement and learner autonomy are crucial to be investigated to have an efficient coherent online learning curriculum.

Technology, a prevailingly used artifact, create variant learning experiences through varying cognitive, emotional and social supports they offer (Binali et al., 2021; Galikyan et al., 2021).This study examines how multimodal and text-based computer mediated conversation modalities (CMCs) affect learner autonomy, learner engagement and learner e-satisfaction.

## Literature review

### Computer mediated conversation modalities and language learning

For their potentials in promoting joint construction of knowledge, computer mediated modalities and platforms are considered as fundamental learning environments (Salloum et al., 2019). Collaboration is a distinctive and necessary component for learning especially in virtual environments (Garrison, 2006). Many learning theories such as social constructivism, activity theory, situated shared cognition have already approved collaborative learning (Munoz-Carril, Hernández-Sellés, Fuentes-Abeledo, & González-Sanmamed, 2021).The connecting potentail of computers can facilitate creation of learning communities that contibute to the construction of shared meaning.

However, different computer modalities have different potentials in promoting effective collaboration. Modality is defined as the medium or channel through which communicative intent is expressed (Pereira (2010). Modalities can be classified with respect to the type of image or temporality of it. With respect to image type, modality is “semiotic realization of one mode” (p. 510), or the way specific information is encoded (e.g., the images transmitted with a webcam in videoconferencing realize a visual modality). Modalities are further classified with respect to temporality of the message such as synchronous which entails simultaneous sending and receiving messages (e.g., when two writers composing in Google Docs simultaneously) and asynchronous in which transmission of the message takes place at different times (e.g., when a writer’s posted texts in weblog are read years later).

Technological and electronic developments have made online CMC equipped with both text and audio modalities (Stockwell, 2007). Modalities have the potential to direct L2 learners' attention to a variety of performance outcomes (Cho, 2018, Colpaert & Spruyt, n.d.). Concerning L2 learning, it is suggested that text chat may help increase students’ verbal participation and increase a higher frequency of student-to-student exchanges since it allows students to overcome their hesitance for learning as the modality is perceived as ‘face-saving’ (Hoffman, 1996) relieving students of their inhibitions and allowing free expression (Freiermuth, 2001) and thus, enhancing students’ willingness to communicate (Freiermuth & Jarrell, 2006). While the majority of studies have focused on text chat as the only verbal communication modality, developments in computer technology made online communication modalities to become multimodal often including text and audio modalities (Stockwell, 2007). It is suggested that multimodal nature of online communication allows more effective collaborative learning than in one dimensional online environment (Dalgarno & Lee, 2010). However, some studies indicated that multimodal nature of online environment may overload learners since they receive both verbal and non-verbal information. For example, the study by Vetter and Chanier (2006) indicated that EFL beginners communicated more than twice as much in the text chat than in the voice chat because they needed to manage both verbal and nonverbal signals at the same time. Some other studies showed positive effects of multimodal CMCs as multimodal online CMC allows more effective collaborative L2 learning than in one-dimensional online modalities (Dalgarno & Lee, 2010) since multimodal entails bodily movements, face tracking, affective sensors (Spikol et al., 2017). The reported inconsistencies on CMC modalities’ efficacy invited further research on their benefits in L2 learning context.

### Computer mediated collaborative writing

Writing skill has always been a challenge for students to acquire since it is postponed till school age. But it has always been an informative subject for researchers because studying it helps to understand developmental processes that cause students choices in prioritizing some aspects of writing over others as a result of moderating factors (Zenouzagh, 2018).

Writing activity has been viewed as a solitary act performed mostly in classroom context. But, constructivist theories of learning and advancement of technology aided learning brought attention to collaborative computer mediated writing (Zenouzagh, 2020). Collaborative writing can be seen as shift from a traditional teacher-directed class to a more interactive and student-centered class where learners actively take part in their class learning (Lin & Maarof, 2013). Collaborative writing is described in terms of social negotiations between several writers in which they construct knowledge and convey it through interaction (Challob et al., 2016). The key concepts related to collaborative learning studies such as learner talk, negotiation of meaning and interaction are revisited by researchers (Twiner et al., 2014). Although computer-mediated writing and its benefits on language development has been extensively examined, online learning modality as a critical component was overlooked (Mohamadi Zenouzagh, 2022).

Informed by social semiotics theory, multimodal writing are texts that communicate meaning via multiple modes of “socially made and culturally shared” semiotic resources (Kress, 2009). The pedagogical value of multimodal writing lies in its ability to expose students to expanded views of literacy with increased semiotic resources beyond language (Shin, Cimasko, Yi, 2020), which epitomizes the communication environment in the digital era. The existence of multiple modes and semiotic resources in multimodal writing leave plenty of room for negotiating of meaning, which is fully realized when learners collaborate with each other to construct multimodal texts. As such, writing is inherently a social activity and technology is the catalyst in this process (Cheung, 2022). While the scope of modality is comprehensive, the scope of discussion in this study will be limited to channels of communication (Multimodal vs. text-based modalities). Although research has shown the benefits of textual and multimodal writing on linguistic aspects of writing such as syntactic complexity (Mohammadi, 2017; Zenouzagh, 2020), little research has considered the potentials of writing modality on other aspects of student learning such as learner autonomy and engagement and satisfaction.

### Learner autonomy and online learning environment

Computer-assisted language learning has been approved to promote learner autonomy as it includes elements of autonomous learning by giving control to students in taking responsibility for their own learning, such as choosing the materials, managing their contact with various genres and types of interaction, often in authentic contexts, and evaluating their own progress, measured through their success in understanding and conveying meanings (Wach, 2012). As the definition suggests, learner autonomy is learners' taking the responsibility of learning at all stages of objective setting, controlling learning processes and setting evaluation criteria (Mohammadi Zenouzagh, 2022). Learner autonomy is a psychological capacity typified as an ability to make decisions requiring capacities of metacognitive knowledge of self, subject and context, knowledge and reflection on learning requiring metacognitive strategies of planning, goal setting, monitoring and evaluation (Van Nguyen & Habók, 2021).

Learner autonomy has taken a center stage as the advancement of technology creates more opportunities for independent learning. Scholars have investigated the enormous potential that different technologies have for autonomous learning where learners can take individual and joint responsibilities with each other for knowledge construction and exercise control on their own learning (Eneau & Develotte, 2012; Ribbe & Bezanilla, 2013). Technologies can also help to train more active learners and expose them to digital, social environments where learners can engage in real world and meaningful interactions with language users. Technologies such as video-conferencing software make geographically separated individuals to communicate in real time. Other related online tools such as discussion forums and online chat environments can help learners engage in social, collaborative and authentic learning opportunities (Zhong, 2018).

Online learning environments require higher levels of learner autonomy among learners. Learner online autonomy requires exhibition of control on monitoring and managing cognitive abilities (Cho & Heron, 2015). It also requires other aspects of learning such as emotion and enjoyment regulation through self, co, and socially regulations to achieve group level engagement in shared regulation processes including joint planning, monitoring, and evaluating (Zhang et al., 2021). A meta-analytic review by Broadbent and Poon (2015) indicated that learners differ in their autonomous learning and the frequencies of their use with strong relation to academic achievement in online classes. The frequently used strategies were metacognitive, time management, critical thinking and effort regulation strategies which were strong predictors of academic success compared to traditional classes. Strategy utilization preferences may also reflect the constraints of learning environment (Broadbent et al., 2021). Online learning may take place with various formats such as synchronous, asynchronous, uni-modal, and multimodal delivery of instruction. This variety may result in different learning experiences (Colson & Hirumi, 2018). This can help teaching practitioners to direct students’ potential to varying instructional objectives (Olsen et al., 2020).

### Learner engagement and online learning environment

Online learning provides learners with ubiquitous learning opportunities and makes the learning processes more learner-centered (Dwivedi et al., 2019). To investigate the effectiveness of online learning, researchers not only use knowledge-based tests to assess learners' academic performances, but also pay more attention to the learners' learning engagement during online learning (Bagheri & Zenouzagh, 2021; Mohamadi, 2017; Wang et al., 2022).

Learner engagement has long intrigued educational researchers due to the inconsistencies in its definition and its measurement (Ayouni et al., 2021). Learner engagement has been conceptualized as students' active involvement in purposeful learning (Buijs & Admiraal, 2013; Galikyan & Admiraal, 2019) and it is seen as the predicting factor in student learning (Guo et al., 2021). Most studies rely on student self-reports and utilize surveys and questionnaires such as National Survey of Student Engagement (Kuh, 2003), Classroom Survey of Student Engagement (Smallwood, 2006) and Student Course Engagement Questionnaire (Handelsman et al., 2005) to tackle student engagement. Research also uses rubrics as instructive tools to measure student engagement through learner experiences and skills participation, emotion and performances (Dixson, 2015; Kahu, 2013).

Most scholars distinguish four dimensions of learner engagement, comprising of behavioral, emotional, cognitive engagement, and social engagement (Bagheri & Zenouzagh, 2021; Deng et al., 2020; Mulia, 2020). In the learning environment, behavioral engagement reflects learning-related activities such as participating in interactions and communications and asking questions. Cognitive engagement is related to mental efforts that learners have to learn a particular skill or acquire intricate knowledge. Emotional engagement deals with students’ positive emotions about their class, peers and teachers, and their online courses (Luan et al., 2020). Relational or social engagement refers to the sense of belonging and the relationships that students develop with their peers, their teachers, and the school. Highly engaged learners are more successful in acquiring knowledge and skills (Martin & Bolliger, 2018). Research confirmed the effect of text-based (Freiermuth & Jarrell, 2006) and multimodal CMC modalities (Collins et al., 2019) on learner engagement.

Research on student engagement in online learning environment mainly focuses on student engagement in uni-modal online learning environment compared to face-to-face classes and thus leaving comparative analysis of student engagement across different online learning environment open to research. Besides, these studies used self-report measures such as surveys and questionnaires in inspecting learner engagement and they fail to unfold the concept of learner engagement from a deeper inspection because learner engagement was investigated using a questionnaire which is subject to risks of reliability of responses and social desirability effect. To fill such a void, the present research investigates the potentials of two online modalities of text and multimodal one in fostering student engagement through conversation analysis techniques of students’ online conversations.

### Learner satisfaction and e-learning environment

The myth of online learning and its acceptance among teachers and learners have been investigated and ascribed to contextual, psychological, social, cultural and demographical factors (Cohen, 2014; Tuyet, 2020). Most of these studies focused on the contextual properties of the learning environment and their effects on acceptance of e-learning environments compared to traditional classes. According to Zhao (2020), unlike most of the dated published studies in the field of education and language learning that investigated success and achievement of the students concerning cliché factors, limited studies published since the start of the COVID-19 pandemic have focused on the particular E-learning related contextual, individual, and psychological variables to determine student satisfaction and acceptance of E-learning courses.

Student satisfaction is a crucial element that contributes to the acceptance and quality of online learning. Students may experience difficulties resulting from wide range of factors such as digital literacy, technical issues, support system and teacher-student interaction. These may result in decreased effectiveness of the curriculum and hence overall student satisfaction. Previous research on different learning environments has suggested a variety of factors affecting user satisfaction with e-learning such as the learner, the curriculum and teacher roles (Sun et al., 2008). However, most of these studies used surveys in which information is organized from researchers’ stand point (Wang, 2003). Besides, in most of these studies, student satisfaction was assessed in one e-learning modality (Kuo et al., 2014) or assessed comparatively with face-to face counterpart (Johnson et al., 2000) and thus leaving comparative investigation of learner e-satisfaction across text-based and multimodal computer assisted learning for research.

### The present study

Most of the studies on CMC are limited to comparisons of online platforms with face-to face classes and therefore, cross comparison between different online modalities is left as potentially an open area for research. Besides, this study contributes and moves the related literature forward since the analysis is built on enacted student performances rather than student self-reports which are subject to reliability issues and inefficacy in depicting related invisible aspects. This study investigates differences between text-based and multimodal computer mediated communication in autonomous learning, learner engagement, and satisfaction. To this end, the following research questions were set to be answered:

1. To what extent do text-based computed mediated writing and multimodal computer mediated writing differ in fostering learner autonomy?
2. To what extent do text-based computer mediated writing and multimodal computer mediated writing differ in fostering learner engagement?
3. To what extent do text-based computer mediated writing and multimodal computer mediated writing differ in fulfilling learner satisfaction?
4. How do text-based computer mediated writing and multimodal computer mediated writing affect students’ writing performance?

## Method

### Research design

This study is a descriptive mix-method study using both quantitative and qualitative data. The dependent variables were learner autonomy, learner engagement, and student satisfaction as well as writing performances. The independent variables were text and multimodal computer-mediated writing modalities. The learner autonomy and student writing performances were investigated by quantitative data collection procedure via questionnaire and student sample writing and the derived data were treated as a test scores. Student engagement was investigated via conversation analysis of transcription of stored conversation in Moodle and discussion logs of text-based forum and chi-square analysis of student engagement dimensions derived from open and axial coding. Student satisfaction was investigated using quantitative thematic analysis of student reflective essaying using Atlasti software.

### Participants

Participants were 40 EFL male and female intermediate Iranian EFL students from English Centers with the age range of 18–23. They were selected based on their proficiency level measured on Oxford placement test and their computer literacy level measured on self-reports on wide range of computer use. They were randomly assigned to multimodal (N = 20), and text based (N = 20) computer mediated communication research groups. Each research group had 5 subgroups of four students. The forming of groups was left to students’ choice. Table 1 shows demographic information of the participants.

**Table 1:** Demographic information of the participants

Number		40
Age		18–23
Inclusion criteria		Language proficiency
Proficiency level		Intermediate
Research group	Text-based	20 (5 groups each including 4 students)
	Multimodal	20 (5 groups each including 4 students)

### Data collection procedure

#### Learner autonomy questionnaire

A Learner autonomy questionnaire developed and validated by Van Nguyen and Habók (2021) was used to measure learner autonomy before and after the research intervention. This questionnaire had 40 items rated on a 5 likert scale tapping into different sub constructs. Table 1 shows information on the attributes of the questionnaire. The reliability of each section of the questionnaire in the present research data set was included in the Table 2.

**Table 2:** Learner autonomy questionnaire

Sub components	Description	Number of items	Examples	Reliability (Cronbach alpha)
Metacognitive skill	Study skills refer to planning, monitoring, and evaluating	15	I reflect on what I learn and look for something important	0.91
Metacognitive knowledge	a) awareness of their strengths and weaknesses in relation to the tasks; (b) an understanding of the tasks they are engaged in; and (c) knowledge of strategies which can help them undertake such tasks”	5	I know my strengths and weaknesses in learning English	0.76
Motivation and desire to learn English	desire is how intensely learners intend to learn English, and complete a learning task	5	I’d like English to be used as much as possible in English class	0.8
Student freedom	The degree to which learners are “permitted” to control their learning	7	I have chances to make suggestions to the teachers	0.84
Beliefs about teacher role	To be aware of the roles of teachers and their own roles because their beliefs as regards their role may strongly influence their exercise of responsibility in or out of class and their readiness to learn English autonomously	8	The teachers should correct all my mistakes	0.82

#### Learner engagement

Studies on theoretical and operational definitions of different dimensions of student engagement have been reviewed to prepare the scheming coding system of student engagement (Table 3) (Guo et al., 2021; Lee & Hannafin, 2016; Silvola et al., 2021; Stephenson et al., 2020). Transcription analysis of stored conversation and text-based log analysis were coded for learner engagement dimension. The coding unit was defined as students’ contributions that show their involvement in the learning task. For example, students actual talk on task rather that procedure talk, less hesitations, less appeal for help as were taken as signs of behavioral engagement. Students’ self or co-regulating learning problems such as coining words to solve communication problem, using metacognitive linguistic knowledge, suggestions for cross check of multiple resources were identified as cognitive engagement codes. Students’ emotional feedbacks to the learning tasks and students’ desire and invitations for group unity and coherence were coded as emotional and social engagements. The beginning and ending of a coding unit is characterized by turns in which any of indicators in coding system in Table 3 could be identified. 849 codes across all transcriptions in two writing modalities were identified. Inter-rater reliability was calculated using Cohen’s kappa (see Appendix A for details).

**Table 3:** Student engagement levels coding

Types	Tokens
Behavioral engagement	Observable behaviors and effort for studies. Active and fluent involvement in learning. Ontime and concentered studying
Cognitive engagement	students’ mental effort to complete tasks using a deep, self-regulated, and strategic approach to learning, rather than superficial learning strategies efforts to formulate questions and inferences, monitor progress. Identification and evaluation of study sources and knowledge construction self-regulated and strategic investment in learning Flexibility and positive coping with challenges on the academic path. Effective use of LA to support one’s learning students’ Exchange of information (External facts, such as sources from websites and articles, and Information and descriptions from teachers, peer students, and the course/task requirement Suggestions for consideration Proposals and calls for time allocation, task allocation, and task procedures etc Connecting and synthesis of ideas from various sources Creating solutions Explicit characterization of message as a solution by participants Vicarious application to real world testing solutions. Providing examples of how problems were solved. Defending solutions Defending why a problem was solved in a specific manner
Emotional engagement	Expressing emotion s, self-disclosure (Expressing vulnerability; include expressions of likes, dislikes, and preferences Expressing personal values, beliefs, and attitudes using emoticons, emojis, stickers repetitious punctuation, repetitious phrases, conspicuous capitalization, emotional attitudes and reactions towards learning willingness to work enthusiastically Expressing compliment and appreciation (complementing others or the contents of others' messages)
Social engagement	Addresses or refers to the group using inclusive (addresses the group as we., us, our, group) Phatics, salutations and greetings (communication that serves a purely social function greetings or closures Social sharing information unrelated to the course.@Vocatives Addressing or referring to the participant by name

#### Learner e-satisfaction

Students’ perceived satisfaction reported in their reflective essays was studied. Coders inspected reflective essays for both positive and negative values that students attributed to either text-based or multimodal CMC modalities. The coding units are students’ statements that indicate students’ self- evaluation and judgments on quality of their learning experience. For example, students in both groups judge different dimensions of their learning experience using adjectives such as “messy, interesting, new adventure, encouraging, confusing”. Open coding procedure was used to identify indicators of student satisfaction and dissatisfaction by two raters. Then, raters used axial coding procedure to construct linkage between codes. The inter-rater reliability was calculated using Cohens’ Kappa (see Appendix B). Student quotations on learner satisfaction dimensions are provided in Table 4.

**Table 4:** Quotations on student satisfaction

		Text-based CMC	Multimodal CMC
Learner dimension	Learners’ attitude	Some times when I write, the dictation checks help me write correctly. I felt I have automated teachers. Sometimes, I become anxious when some people misinterpret my text-based messages	It was much easier to follow class discussions
	Learner internet self-efficacy	I easily used the website	I am confident at navigating in internet
Teacher dimension	Instructor presence	Students do not do their share when they write their homework because the teacher was not online. But later the teacher entered our group by chance and students afraid and did their share	It was a good feeling to teachers’ face too. In our work, the teacher controlled performances by being an occasional guess
	Instructor digital competences	The teacher introduced some solutions when we have problems with the website. Sometimes, the website could not be reached	The instructor helps me give permission to Firefox to active my microphone and webcam. At the beginning everything was difficult but now we learned everything about online learning
Internet dimension	Internet quality	At the beginning of the class, because all students try to get connected, the site was out of reach for me and after a long delay when I enter the class the teacher penalized me	I am sick and tired of online classes only because of internet speed. The videos and video are frizzed. When we inform the teacher, he says nothing can be done and it is related to internet infra structures
	Support system	Every time I call Ms. A to tell my problem, I was in waiting list	I call support system, but with no exception, my calls were not received and I left behind the class
Curriculum dimension	e-learning course flexibility	When we do our homework on line, we set a time ourselves and this is really convenient for us. Most of the time, we set the time at night, when the quality of internet is better	we could have make up sessions at our convenience and since the sessions were recorded we could access the sessions when we could not participate in the class
	Course quality		
	Flexibility in interaction	My group can interact with each other very easily; we could follow each other’s sentences because we selected different colors in website. We could also ask the teacher to change our team if the team does not work	We could follow each other’s’ discussion easily
	Support system	If the university provides more support, I prefer online classes than face-to-face, we can save more time	A supporting agency should be an important component of online classes, otherwise students may feel confused

### Experimental procedure

Students were asked to write their assignments collaboratively in two online contexts, i.e., text-based CMC group and multimodal CMC group. They participated in a tutorial session to get familiarized with Moodle and Writing Forum platforms, to receive their log-in credentials, and details on the structure of collaborative writing assignment. To assure consistency across groups, everything in both groups were kept constant including the writing tasks and topics, teachers’ explicit teaching and teachers’ scoring method. In both groups, the following procedure was taken: 1) the students chose their partner as they wished; (2) the teachers had the students do brainstorming about the writing topics; (3) the students researched and gathered information using different sources; (4) the students wrote the outline and gave it back to the teacher and the teacher provided pertinent comments; (5) then the students planned and wrote the first draft; (6) the students checked out the first draft according to the checklist provided by the teacher in advance; (7) each student edited the essay individually with different highlight colors so that when they handed in their essays together, they could track each other’s ideas and provide justifications for the required revisions; (8) students handed in their writing to the teacher and the teacher commented on the language, content, and organization of their writings; (9) and finally, students received the teacher’s comments and revised the paper together. Sample student collaborative writing is provided in Appendix C.

Modular object-oriented dynamic learning environment (Moodle) was used as a multimodal CMC. This platform allows synchronous chats and a synchronous discussion for geographically separated users. In Moodle, teachers and students can collaborate in both voice and video modalities, send, and respond files and comments, and can use the tracking and feedback system. Moodle has a wide range of ways in which people can create representations of their knowledge and share them. The course structure itself is a terrific way to construct a shared and active representation of the learning journey that everyone is going through Forums of course are the core of this, providing spaces for discussion and sharing of media and documents (using the media plugin filters, attachments or simply links). Wikis are collaboratively built pages useful for group work and other negotiations. Glossaries are collaboratively built lists of definitions that can then appear throughout the course. Databases are an extension of this idea allowing participants to enter structured media of any type (for example a collection of digital photos or a library of references). The Recent Activity block shows a great deal of information about what has happened recently, and via link you can see reports with more detail. Things that happened include changes to the course and forum posts.

Students in multimodal CMC group used Moodle to negotiate and collaborate as they were doing writing assignments. Students in this group shared the ideas on the topic of the writing task, generated ideas through negotiation with each other and wrote down their first draft e-collaboratively through Skype as audio conference writing. They negotiated and collaborated on the first draft until they reached shared understating on the final draft. Students were informed about how to work with Moodle.

An e-writing forum was launched on http//e-writingforum.ir by the researchers. As a text-based CMC, students used writing, commenting, and responding to comments options of the forum and provided comments on each other’s writing performance. Participants were required to write a writing assignment collaboratively. Students were instructed to create accounts on the website. Students were paired on the website. Students logged into their accounts on the forum and entered the section in which they were grouped with their partners. They were asked to log in to the forum and find the thread posted by the teacher and to post their ideas and respond to their group mate’s ideas via the options available at the tool bar of the forum. The toolbar included options for posting an idea, editing a post, providing comments, replying to the comments, and replying with quotes.

At the end of the treatment, students were asked to write a reflective essay on the extent to which they are satisfied with the learning environment they experienced during the treatment. Students’ first and last writing performances during 14 sessions of treatment was also compared to investigate which CMC modality had higher potentials in improving students’ writing quality.

### Data analysis procedure

To investigate to what extent text-based and multimodal computer mediated writing differ in learner autonomy, students’ responses were treated as test scores adding to their level of learner autonomy. The mean scores were compared using independent sample t-test. Via open and axial coding of transcribed conversations in Moodle and discussion logs in text-based forum, researchers identified student behavioral, cognitive, and emotional engagement dimensions. The chi-square analysis of the identified codes and categories was used to investigate the extent to which text-based and multimodal computer mediated writing differed in promoting learner engagement. Moreover, a one-way analysis of covariance (ANCOVA) was run to compare the multimedia and text-based groups’ means on posttest of writing performance after controlling for the effect of pretest. Besides, the codes derived from the reflective essays were discussed in relation to students’ reflective quotations to indicate on what grounds multimodal and text-based CMCs satisfied students.

## Results

### Learner autonomy in text-based and multimodal computer mediated writing

An independent-samples t-test was run to compare the multimedia and text-based groups’ means on autonomy to probe the first research question. Table 5 displays the results of the descriptive statistics for the two group on autonomy. The results indicated that the text-based group (M = 163.35, SD = 27.93) had higher mean than the multimedia group (M = 135.80, SD = 35.96) on autonomy.

**Table 5:** Descriptive statistics; autonomy by groups

Group	N	Mean	Std. Deviation	Std. Error Mean
Multimodal	20	135.80	35.967	8.043
Text-Based	20	163.35	27.937	6.247

Table 6 displays the results of the independent-samples t-test. It should be noted that the assumption of homogeneity of variances was retained. As displayed in Table 6, the non-significant results of the Levene’s test (F = 3.91, p > 0.05) indicated that the two groups were homogenous in terms of their variances on autonomy. That was why the first row of Table 6; i.e., “Equal variances assumed” was reported. The results of independent samples t-test; (t (38) = 2.70, p < 0.05, r = 0.401 representing a moderate effect size^1^) indicated that the text-based group had a significantly higher mean than the multimedia group on autonomy.

**Table 6:** Independent-samples t-test; autonomy by groups

	Levene's Test for Equality of Variances					t-test for Equality of Means			
	F	Sig	T	Df	Sig. (2-tailed)	Mean Difference	Std. Error Difference	95% Confidence Interval of the Difference	
								Lower	Upper
Equal variances assumed	3.918	0.055	2.705	38	0.010	27.550	10.184	6.934	48.166
Equal variances not assumed			2.705	35.808	0.010	27.550	10.184	6.893	48.207

### Learner engagement text-based and multimodal computer mediated writing

To investigate significant differences between the frequencies of student engagement dimensions of behavioral, cognitive, emotional, and social by multimedia and text-based groups, chi-square test was used. Table 7 displays the frequencies, percentages, and standardized residuals (Std. Residual) for the two groups. It should be noted that any Std. Residual higher than ± 1.96 indicates that the frequency is significant beyond/below what was expected.

**Table 7:** Frequencies, percentages and standardized residuals; types of engagement by groups

		Engagement				Total
		Behavioral	Cognitive	Emotional	Social	
Multimodal	Count	100	30	80	180	390
	% within Group	25.6%	7.7%	20.5%	46.2%	100.0%
	Standardized Residual	-3.2	-3.8	3.4	3.6	
Text-Based	Count	200	100	39	120	459
	% within Group	43.6%	21.8%	8.5%	26.1%	100.0%
	Standardized Residual	3.0	3.5	-3.2	-3.3	
Total	Count	300	130	119	300	849
	% within Group	35.3%	15.3%	14.0%	35.3%	100.0%

The results indicated that text-based group employed behavioral (43.6%, Std. Residual = 3 > 1.96) and cognitive (21.8%, Std. Residual = 3.5 > 1.96) engagement strategies significantly more than the multimedia group whose results were (25.6%, Std. Residual = -3.2 > -1.96) for behavioral engagement, and (7.7%, Std. Residual = -3.8 > -1.96) for cognitive engagement. On the other hand, multimedia group employed emotional (20.5%, Std. Residual = 3.4 > 1.96) and social (46.2%, Std. Residual = 3.6 > 1.96) engagement strategies significantly more than the text-based group whose results were (8.5%, Std. Residual = -3.2 > -1.96) for emotional engagement, and (26.1%, Std. Residual = -3.3 > -1.96) for social engagement.

**Fig. 1:**
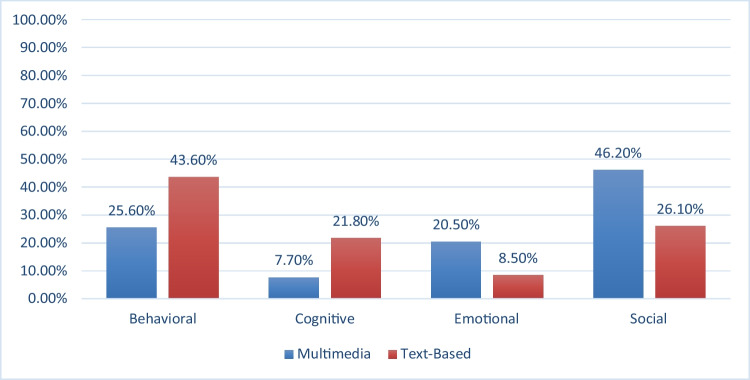
Percentages of engagement strategies by groups

Table 8 displays the results of the analysis of chi-square. The results (χ^2^ (3) = 92.15, p < 0.05) indicated that there were significant differences between the multimedia and text-based groups’ use of engagement strategies. The results also were indicated in Fig. 1. As it was discussed above, the multimedia group employed emotional and social strategies significantly more than the text-based group, while the latter group used behavioral and cognitive strategies significantly more than the multimedia group. The effect size for the chi-square was 0.329 which represent a moderate effect size (Cramer’s V = 0.329 representing a moderate effect size^2^) (Gray and Kinnear, 2012).

**Table 8:** Chi-square tests; types of engagement by groups

	Value	df	Asymptotic Significance (2-sided)
Pearson Chi-Square	92.153^a^	3	0.000
Likelihood Ratio	94.632	3	0.000
Linear-by-Linear Association	61.982	1	0.000
N of Valid Cases	849		
Cramer’s V	0.329		0.000

a. 11 cells (68.8%) have expected count less than 5. The minimum expected count is 54.66.

### Learner satisfaction in text-based and multimodal CMC writings

**Fig. 2:**
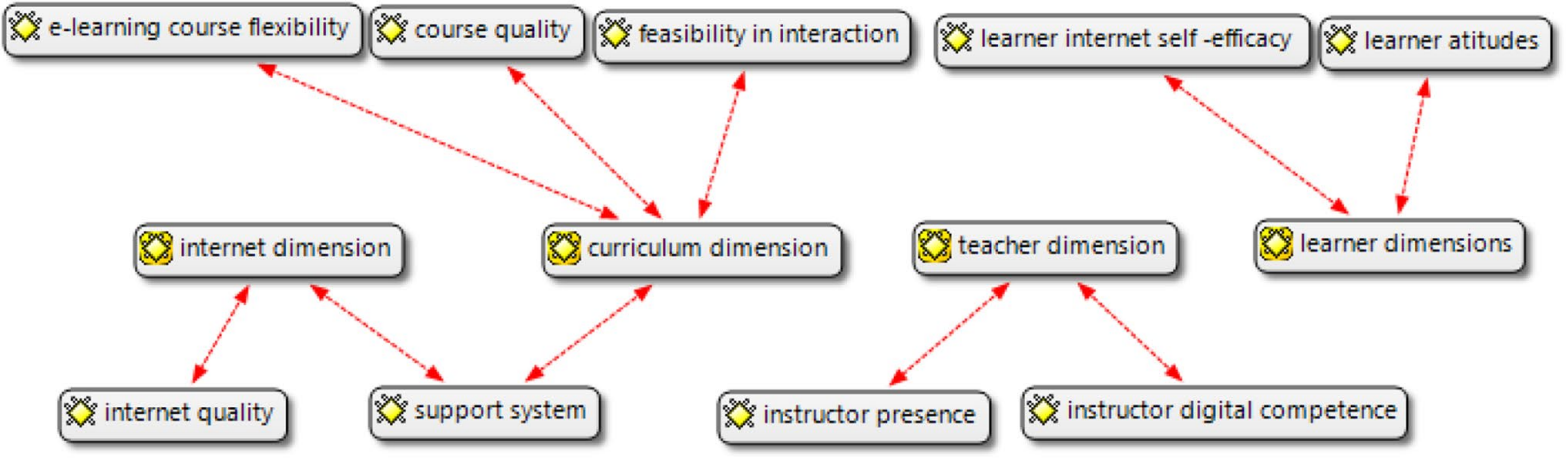
Learning e-satisfaction dimensions

Learner satisfaction about learning experiences in two CMC modalities were studied via theme analysis of student reflective essays and coded using open and axial coding procedure to categorize the themes. Student’ satisfaction analysis on 258 quotations in 40 reflective essays of both groups resulted in identification of four categories of codes including *learner dimension* (learners’ attitude, learner internet self-efficacy), *teacher dimension* (teacher presence, teacher digital competences), *curriculum dimension* (curriculum flexibility, course quality, flexibility in interaction support system) and *internet dimension* (internet quality and support system) (Fig. 2). Only internet dimension received negative judgments from students of both groups. The quotations from student reflective essays are provided in Table 11 in Appendix D.

### Text-based and multimodal computer mediated writing performances

A one-way analysis of covariance (ANCOVA) was run to compare the multimodal and text-based groups’ means on posttest of writing performance after controlling for the effect of pretest. Required assumptions for ANCOVA were checked. One-way ANCOVA has three assumptions, i.e., homogeneity of variances of groups, linearity, and homogeneity of regression slopes. First, t the assumption of homogeneity of variances was retained on posttest of writing test (F (1, 38) = 0.925, p < 0.05) (Table 11, Appendix D). Second, one-way ANCOVA assumes that there is a linear relationship between dependent variable (posttest of writing performance) and covariate (pretest). The linearity assumption was retained, i.e. (F (1, 39) = 12.52, p < 0.05, η^2^ = 0.288 representing a large effect size^3^) (Table 12, Appendix E). And finally; one-way ANCOVA assumes that the linear relationship between pretest and posttest are roughly equal across the two groups; homogeneity of regression slopes. The non-significant interaction between covariate (pretest) and independent variable (types of treatment); i.e. (F (1, 36) = 0.601, p > 0.05, Partial η^2^ = 0.016 representing a weak effect size^4^) was retained (Table 13 in Appendix F). Table 9 displays the descriptive statistics for the multimedia and text-based groups on posttest of writing performance after controlling for the effect of pretest. The results showed that the text-based group (M = 7.51, SE = 0.113) had a higher mean than the multimedia group (M = 6.75, SE = 0.113) after controlling for the effect of pretest.

**Table 9:** Descriptive statistics; posttest of writing performance by groups with pretest

Group	Mean	Std. Error	95% Confidence Interval	
			Lower Bound	Upper Bound
Multimodal	6.758^a^	0.113	6.528	6.988
Text-Based	7.517^a^	0.113	7.287	7.747

a. Covariates appearing in the model are evaluated at the following values: Pretest = 5.10.

Table 10 displays the main results of one-way ANCOVA. The results (F (1, 37) = 22.38, p < 0.05, partial η^2^ = 0.377 representing a large effect size) indicated that the text-based group significantly outperformed the multimedia group on the posttest of writing performance after controlling for the effect of pretest.

**Table 10:** Tests of between-subjects effects; posttest of writing performance by groups with pretest

Source	Type III Sum of Squares	df	Mean Square	F	Sig	Partial Eta Squared
Pretest	5.722	1	5.722	22.252	0.000	0.376
Group	5.755	1	5.755	22.380	0.000	0.377
Error	9.515	37	0.257			
Total	2058.250	40				

## Discussion

This study aimed at investigating the comparative potential of text-based and multimodal learning modalities. The results indicated that students were more autonomously, behaviorally, and cognitively engaged in text-based modality and more emotionally and socially engaged in multimodal modality. Students reported similar value judgments as far as student satisfaction is concerned. The results revealed that different modalities have different potentials in directing student learning.

### Learner autonomy in text-based and multimodal computer mediated writing environments

As far as learner autonomy is concerned, text-based modality has shown higher potential in increasing learner autonomy. The most probable justification for superiority of text-based modality in fostering learner autonomy is that, students experience more cognitive presence which is achieved through learners’ sustained reflection and discussion in meaning construction (Janssona et al., 2021). Cognitive presence is characterized by triggering event, Exploration, Integration, and Resolution which are key to autonomous learning (Garrison, 2006). The findings align with many studies on the association between text- based computer assisted writing and learner autonomy. For example, the experimental study by Pathan et al. (2021) indicated that text blogging has affected engineering higher education students’ learner autonomy. Research also supported that discussion forums and online text chat environment engaged learners in more collaborative opportunities in which students jointly take the responsibility of learning (Zhong, 2018). Research also indicated that in text-based computer mediated writing, students use more metacognitive and effort regulation strategies that are predictors of autonomous learning, (Broadbent et al., 2021). The results of this study also show contradictions with the existing literature on learner autonomy and online learning. For example, Garrison et al. (2001) indicated that multimodal learning led to more autonomous learning through establishing motivation, creativity and collaboration. Other studies also indicated that multimodal learning results in more autonomous learners since learners’ competencies in interpreting, employing, and interacting with various semiotic resources, of which language is just one were involved. Interactions in multimodal platforms direct more authentic learning context and engage learners more in co-construction of knowledge and cognitive control of learning (Liu & Moeller, 2019). Several other studies reported impact of multimodal learning on learner autonomy (Hafner & Miller, 2011; Seeger, 2019; Villamizar & Mejía, 2019); all confirming positive role. However, they were compared with the traditional campus-based learning.

### Learner engagement in text-based and multimodal computer mediated writing environments

As far as student engagement was concerned, the result of the present research indicated that text-based CMC group outperformed multimodal CMC group in terms of behavioral and cognitive engagement. Yet multimodal CMC group reported higher emotional and social engagement. Why students are more socially and emotionally engaged in multimodal learning can be explained by student’s social presence defined as participants’ tendency to identify, develop inter-personal relationships and communicate with the community (e.g., course of study), in a trusting environment (Janssona et al., 2021). Social presence is characterized by personal/affective category, open communication and group cohesion which are key to student engagement (Garrison & Akyol, 2015). The results corroborate several studies which supported the impact of text-based learning modality on learner engagement and autonomy. For example, the results of the study by Pineda-Báez et al. (2019) indicated that in text-based CMC, students were more cognitively engaged whereas in visual CMC, students were more socially engaged. The superiority of text-based modality in more engaging learners compared to oral modalities were also confirmed in research by Traphagan et al. (2010). Similarly, Swartzwelder et al. (2019) indicated that students felt more engaged and interactive in text-based discussion compared to video-based discussions with native speakers. The results of the present study contradict the existing literature on multimodal learning and learner engagement. Whereas the present research approved higher potential in text-based modality in engaging students compared to multimodal learning, there are several studies that approved the opposite, For example, research indicated more cognitive engagement in multimodal learning compared to text-based modalities (Sankey et al., 2010). Likewise, the study by Taylor and Huang (2011) confirmed the superiority of voice based threads compared to text and video threads in engaging students.

### Writing quality in text-based and multimodal computer mediated writing environments

As far as the quality of writing was concerned, results of this study indicated that both text- based and multimodal CMCs optimized higher quality writing. The results are in accord with several other studies. Similarly, scholars such as Jepson (2005) and Satar and Özdener (2008) stated that both multimodal and text- based CMCs have impacts on optimizing student learning. However, it is assumed that errors in spoken form are often ignored for the sake of fluent interaction whereas in written form they have a more permanent nature and this may put text based CMCs in advantage over multimodal CMCs (Sampson, 2012).

### Student e-satisfaction in text-based and multimodal computer mediated writing environments

With respect to student satisfaction, the results indicated that students’ satisfaction was affected by factors such as *learner dimension* (learners’ attitude, learner internet self-efficacy), *teacher dimension* (teacher presence, teacher digital competences), *curriculum dimension* (curriculum flexibility, course quality, flexibility in interaction support system) and *internet dimension* (internet quality and support system) reflected students’ e-satisfaction in both groups. However, the designers of e-learning systems are called to combine different analytical and/or stochastic methods in assessing degree of customers’ expectations and their level of satisfaction. A holistic approach based on users’ satisfaction level and the appropriate measurement analysis should give support to the designers in improving existing and designing new more attractive web-based learning models in the contemporary educational blended (Bauk et al., 2014).

## Conclusion and implications

This study indicated that text-based and multimodal CMCs have different potentials in engaging students, fostering autonomous learning and in turn resulting in learner satisfaction. The results of between group comparison indicated that students were more autonomous in text-based modality than in multimodal CMC. Chi-square analysis indicated that text-based CMC group outperformed multimodal CMC group in terms of behavioral and cognitive engagement. Yet, multimodal CMC group reported higher emotional and social engagement. One-way ANCOVA results also indicated the students in text-based CMC group outperformed Multimodal CMC group in terms of writing quality. Learner e-satisfaction was examined by network mapping of open codes of student reflective essays. Four categories were identified to reflect students' e-satisfaction: a) *learner dimension* (including learners’ attitude, learner internet self-efficacy), b) *teacher dimension* (including teacher presence, teacher digital competences), c) *curriculum dimension* (including curriculum flexibility, course quality, flexibility in interaction support system) and d) *internet dimension* (including internet quality and support system) reflected students’ e-satisfaction. Only internet dimension received negative judgments from students of both groups. As indicated in this research, the learning environments that foster real time presence to create an atmosphere of trust, feedback and openness among students may increase learner satisfaction (Sharifrazi & Stone, 2019).

Theoretically speaking, the present research findings imply that dual function for social means for communication and cognitive means for co-regulations towards co-regulated learning are deemed highly valuable for L2 development. However, dynamics of interaction is influenced by the mode of interaction. This study also suggests that the multimodal CMCs are deemed as a site with varying levels of functionality in engaging students in autonomous learning.

Practical implications of the findings suggest that teachers can implement text-based CMC when real time on task learner behavior is needed because it engages learners more in actual performance. Multimodal CMC can be implemented when scaffolding through emotions and collaborations is needed. Multimodal CMCs allow richer cues, instant feedback, and use of natural languages. However, text-based CMC users circumvent the constrains of the channel (lack of nonverbal cues) to achieve their communicative goal. Multimodal CMC users may turn off videos which may give this feeling of teaching to voids. Besides, the video options may affect students ‘optimal performance due to their touch on learner self- confidence. This may give an advantage to text-based CMCs as they may contribute to student self-disclose. Text-based CMC can improve teaching quality in terms of creating opportunities for interpretive communication skills and multimodal CMC can enhance direct interpersonal and presentational skills. As far as assessment and measurement are concerned, the text-based modality can better help examiners to evaluate individual learning. And since multimodal learning is more open to student social and emotional engagement, this modality can be more useful for assessment of collaborative performances. The results of student satisfaction imply that irrespective of communication modality, student satisfaction is integral to successful learning. Results also imply that text-based communication modality can better assist writing teachers than multimodal communication modality.

The findings of the research are limited in several ways. First, learner autonomy was assessed in summative fashion via questionnaire. Future research can use formative assessment to assess invisible aspects that could not be detected through summative assessment techniques such as how learning autonomy evolves in different learning contexts. Besides, responses to the questionnaire might be affected by social desirability effect. Future research is needed to design techniques that can minimize this effect. In addition, since student group work skills and individual accountability might affect student performances, future research can assess interactive CMCs that foster group unity and cater for individual accountability and in turn maximize learning opportunities.

## Data Availability

Atlasti and SPSS entry were used.

## Appendix A

Displays the Cohen’s Kappa inter-rater reliability indices for the two raters who rated the four types of engagement. The results showed that there were significant agreements between the two raters on the;

- Behavioral engagement (Kappa = 0.834, Z = 5.40, p < 0.05),
- Cognitive engagement (Kappa = 0.826, Z = 5.42, p < 0.05),
- Emotional engagement (Kappa = 0.877, Z = 5.71, p < 0.05), and
- Social engagement (Kappa = 0.844, Z = 5.40, p < 0.05).

### Cohen’s Kappa Inter-Rater

**Table:**

Engagement	N	Minimum	Maximum
Behavioral	0.834	5.40	0.000
Cognitive	0.826	5.42	0.000
Emotional	0.897	5.71	0.000
Social	0.844	5.40	0.000

## Appendix B

The following table displays the Cohen’s Kappa inter-rater reliability indices for the two raters who rated the essays using four codes. The results showed that there were significant agreements between the two raters on the;

- Learner dimension (Kappa = 0.835, Z = 5.29, p < 0.05),
- Teacher dimension (Kappa = 0.849, Z = 5.39, p < 0.05),
- Curriculum dimension (Kappa = 0.747, Z = 4.77, p < 0.05), and
- Internet dimension (Kappa = 0.750, Z = 4.75, p < 0.05).

### Cohen’s Kappa Inter-Rater

**Table:**

Codes	N	Minimum	Maximum
Learner dimension	0.835	5.29	0.000
Teacher dimension	0.849	5.39	0.000
Curriculum dimension	0.747	4.77	0.000
Internet dimension	0.750	4.75	0.000

## Appendix C

Shots of student writing in online classes.

## Appendix D

Table 11

**Table 11:** Levene's Test of Equality of Error Variances; Posttest of Writing Performance

F	df1	df2	Sig
0.925	1	38	0.342

Tests the null hypothesis that the error variance of the dependent variable is equal across groups.

## Appendix E

Table 12

**Table 12:** Testing Linearity of Relationship between Pretest and Posttest of Writing Performance

			Sum of Squares	df	Mean Square	F	Sig
Posttest * Pretest	Between Groups	(Combined)	5.899	4	1.475	3.537	0.016
		Linearity	5.223	1	5.223	12.526	0.001
		Deviation from Linearity	0.676	3	0.225	0.540	0.658
	Within Groups	14.595	35	0.417			
	Total	20.494	39				
Eta Squared		0.288					

## Appendix F

Table 13

**Table 13:** Testing Homogeneity of Regression Slopes; Writing Performance

Source	Type III Sum of Squares	df	Mean Square	F	Sig	Partial Eta Squared
Group	0.022	1	0.022	0.085	0.773	0.002
Pretest	5.736	1	5.736	22.064	0.000	0.380
Group * Pretest	0.156	1	0.156	0.601	0.443	0.016
Error	9.359	36	0.260			
Total	2058.250	40				

## Footnotes

1 The r-effect size should be interpreted based on these criteria; .10 = Weak, .30 = Moderate, and .50 = Large (Field 2018). The r effect size was computed using the following formula; $r=\sqrt{\frac{t^2}{t^2+df}}$ (Field 2018, p 609).

2 Cramer’s V = square root of chi-square / total frequency; i.e. Square root of 92.153 / 849 = .329. Cramer’s V “may be interpreted in a manner similar to a correlation” (George and Mallery 2020, p 372). That is to say; .10 = Weak, .30 = Moderate and .50 = Large.

3 Eta Squared was computed as Sum of Squares Between Groups / Sum of Squares Total; and should be interpreted using these criteria, .01 = Weak, .06 = Moderate, and .14 = Large (Gray and Kinnear 2012, p 244; Field 2018, p 737).

4 Partial Eta Squared should be interpreted using the following criteria; .01 = Weak, .06 = Moderate, and .14 = Large (Gray and Kinnear 2012, p 323; and Pallant 2016, p 285).

## Data Availability

The data is available on request by emails.

## Declarations

### Data deposition

Data is available for submission if required through anonymous email.
